# In Vitro Analysis of *N*-Nitrosodimethylamine (NDMA) Formation From Ranitidine Under Simulated Gastrointestinal Conditions

**DOI:** 10.1001/jamanetworkopen.2021.18253

**Published:** 2021-06-28

**Authors:** Zongming Gao, Michael Karfunkle, Wei Ye, Tim Andres Marzan, Jingyue Yang, Timothy Lex, Cynthia Sommers, Jason D. Rodriguez, Xiaomei Han, Jeffry Florian, David G. Strauss, David A. Keire

**Affiliations:** 1 Division of Complex Drug Analysis and Division of Pharmaceutical Analysis, Office of Testing and Research, Office of Pharmaceutical Quality, Center for Drug Evaluation and Research, Food and Drug Administration, St Louis, Missouri; 2 Division of Applied Regulatory Science, Office of Clinical Pharmacology, Office of Translational Sciences, Center for Drug Evaluation and Research, Food and Drug Administration. Silver Spring, Maryland

## Abstract

**Importance:**

A publication reported that *N*-nitrosodimethylamine (NDMA), a probable human carcinogen, was formed when ranitidine and nitrite were added to simulated gastric fluid. However, the nitrite concentrations used were greater than the range detected in acidic gastric fluid in prior clinical studies.

**Objective:**

To characterize NDMA formation following the addition of ranitidine to simulated gastric fluid using combinations of fluid volume, pH levels, and nitrite concentrations, including physiologic levels.

**Design, Setting, and Participants:**

One 150-mg ranitidine tablet was added to 50 or 250 mL of simulated gastric fluid with a range of nitrite concentrations from the upper range of physiologic (100 μmol/L) to higher concentrations (10 000 μmol/L) with a range of pH levels. NDMA amounts were assessed with a liquid chromatography–mass spectrometry method.

**Main Outcomes and Measures:**

NDMA detected in simulated gastric fluid 2 hours after adding ranitidine.

**Results:**

At a supraphysiologic nitrite concentration (ie, 10 000 μmol/L), the mean (SD) amount of NDMA detected in 50 mL simulated gastric fluid 2 hours after adding ranitidine increased from 222 (12) ng at pH 5 to 11 822 (434) ng at pH 1.2. Subsequent experiments with 50 mL of simulated gastric fluid at pH 1.2 with no added nitrite detected a mean (SD) of 22 (2) ng of NDMA, which is the background amount present in the ranitidine tablets. Similarly, at the upper range of physiologic nitrite (ie, 100 μmol/L) or at nitrite concentrations as much as 50-fold greater (1000 or 5000 μmol/L) only background mean (SD) amounts of NDMA were observed (21 [3] ng, 24 [2] ng, or 24 [3] ng, respectively). With 250 mL of simulated gastric fluid, no NDMA was detected at the upper physiologic range (100 μmol/L) or 10-fold physiologic (1000 μmol/L) nitrite concentrations, while NDMA was detected (mean [SD] level, 7353 [183] ng) at a 50-fold physiologic nitrite concentration (5000 μmol/L).

**Conclusions and Relevance:**

In this in vitro study of ranitidine tablets added to simulated gastric fluid with different nitrite concentrations, ranitidine conversion to NDMA was not detected until nitrite was 5000 μmol/L, which is 50-fold greater than the upper range of physiologic gastric nitrite concentrations at acidic pH. These findings suggest that ranitidine is not converted to NDMA in gastric fluid at physiologic conditions.

## Key Points

**Question:**

Does ranitidine convert to *N*-nitrosodimethylamine (NDMA), a probable human carcinogen, in simulated gastric fluid at physiologic pH levels and nitrite concentrations?

**Findings:**

In this in vitro study of 150-mg ranitidine tablets added to simulated gastric fluid, NDMA did not form when gastric nitrite concentrations were at the upper range of physiologic or at nitrite concentrations as much as 50-fold greater than the upper range.

**Meaning:**

In this study, 150-mg ranitidine tablets did not convert to NDMA in simulated gastric fluid with physiologic nitrite concentrations.

## Teaser

This study characterizes *N*-nitrosodimethylamine (NDMA) formation following the addition of ranitidine to simulated gastric fluid using combinations of fluid volume, pH levels, and nitrite concentrations, including physiologic levels.

## Introduction

Ranitidine is a histamine_2_ (H2) receptor antagonist that inhibits gastric parietal cell acid secretion for the treatment of gastroesophageal reflux and peptic ulcer disease.^1^ The ranitidine drug product, sold under the brand name Zantac, was approved for human use in 1983 and became a widely used stomach acid inhibitor with more than 18 million prescriptions written in 2018.^2^ However, many countries withdrew ranitidine drug products from their markets after *N*-nitrosodimethylamine (NDMA), a probable human carcinogen, was detected and observed to increase over time under normal storage conditions to amounts greater than the acceptable daily intake.^3,4,5,6^

In addition, ranitidine was proposed to convert to NDMA in vivo, as described in an article by Braunstein et al titled “Analysis of Ranitidine-Associated *N*-Nitrosodimethylamine Production Under Simulated Physiologic Conditions.”^7^ However, that article did not provide any justification for the physiologic relevance of the nitrite (NO_2_) concentrations studied (1000 to 50 000 μmol/L). The proposed mechanism for ranitidine^8^ to form NDMA involves 2 separate nitrite-dependent steps, and the reaction rate for the second step is proportional to the concentration of the NDMA precursor dimethylamine and the square of the concentration of protonated nitrite.^9^ Thus, pH level, ranitidine concentration, and nitrite concentration are key reaction conditions that will affect whether NDMA will be formed.

Review of the literature revealed that 25 μmol/L of nitrite (ie, 40-fold lower than the lowest concentration studied by Braunstein et al^7^) had been considered the upper range of normal in the acidic fasting stomach based on published clinical studies.^10^ However, that assessment was performed in 1985, and an updated evaluation of physiologic nitrite concentrations was warranted, with additional consideration of the potential effect of food; of medications, such as H2 blockers and proton pump inhibitors; and among specific patient groups. The current in vitro study characterized the potential formation of NDMA following the addition of ranitidine to simulated gastric fluid using different combinations of fluid volume, pH, and nitrite concentration, which included physiologic levels.

## Methods

Physiological boundary conditions for in vitro studies on ranitidine were based on previous clinical studies. In vitro experiments were performed by adding 150-mg ranitidine tablets to simulated gastric or intestinal fluid with varying pH, fluid volume, and nitrite concentration. This study did not involve patients; thus, it was not under the purview of an institutional review board. The eMethods in the Supplement describe materials used.

### Selecting Boundary Conditions for Physiologic Gastric Nitrite Concentrations

eFigure 1 and eTable 1 in the Supplement summarize information from 26 clinical studies reporting simultaneous measurements of gastric nitrite concentration and pH from a variety of patient populations, receiving different medications, and tested in fasted and fed states. Most studies were published in the 1970s or 1980s, when the nitrite content in many foods was significantly higher than today and in an era before proton pump inhibitors and treatment for *Helicobacter pylori*. The more limited medical treatment options led to a much higher rate of surgical treatment for ulcers, which altered gastric anatomy and physiology, resulting in higher gastric nitrite concentrations. In addition, most studies measured nitrite concentration with the Griess reaction, which was described in the nineteenth century and does not match the sensitivity or accuracy of newer technologies. With this context, certain studies are of higher quality than others, and analytical measurement error or imprecision may contribute to individual patient outliers. Studies using improved analytical methods and nitrite data by 1-unit pH increases are highlighted.

### Fasting Gastric Nitrite and pH Across Diverse Patient Populations

**Figure 1.**
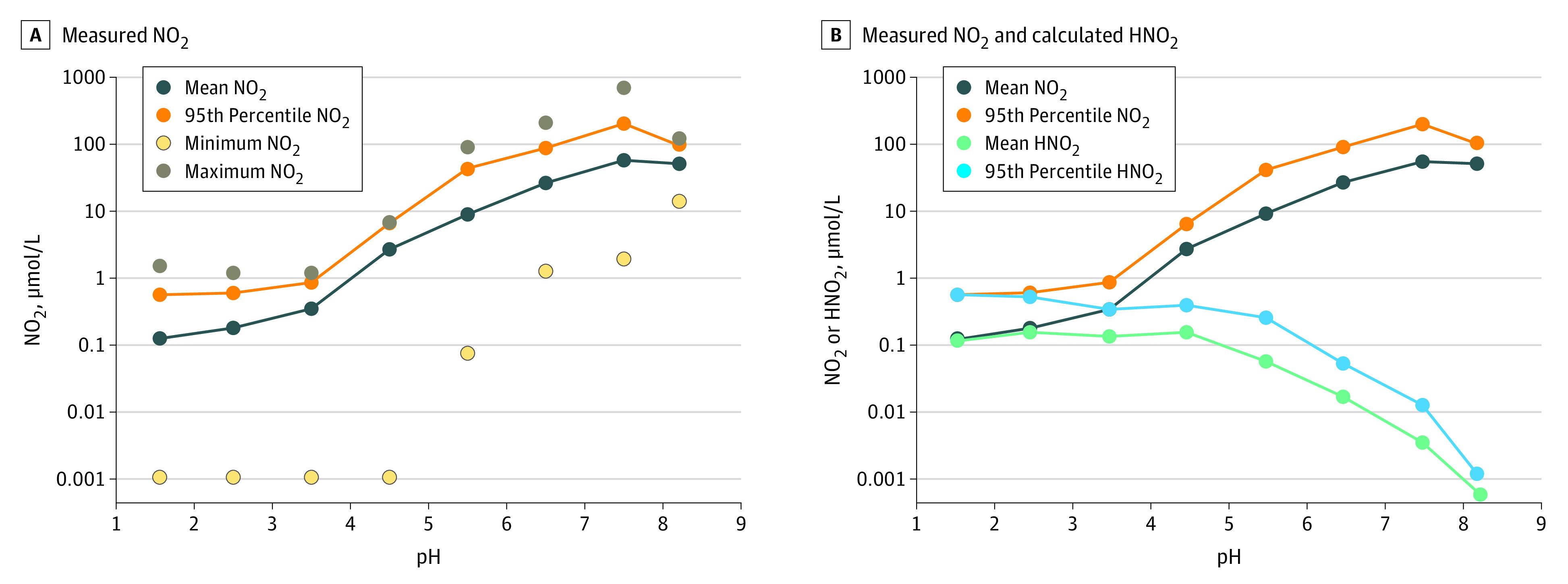
pH Dependence of Nitrite (NO_2_) Concentration From the Clinical Data in the Study by Xu and Reed^11^ Plots of the nitrite concentration in gastric fluid from Xu and Reed^11^ from 457 patients undergoing upper endoscopy for various gastrointestinal conditions. The patients included those with chronic superficial gastritis, chronic atrophic gastritis, gastric ulcer, gastric carcinoma, pernicious anemia, esophagitis, duodenal ulcer, and normal gastric histology and those who had undergone vagotomy or partial gastrectomy. Points are displayed at the midpoint of the pH range for each group. The actual pH ranges of each group are 1.13 to 1.99, 2.00 to 2.99, 3.00 to 3.99, 4.00 to 4.99, 5.00 to 5.99, 6.00 to 6.99, 7.00 to 7.99, and 8.00 to 8.42. See eTable 1 in the Supplement for additional details.

In 1993, Xu and Reed^11^ developed an improved nitrite analytical method and published the largest series of patients (n = 457) undergoing upper endoscopy for various gastrointestinal conditions. Figure 1A shows the reported minimum, maximum, and mean nitrite concentrations as well as the calculated 95th percentile of the population for each 1-unit increment of pH.^11^ Nitrite concentration increased with pH. The 95th percentile nitrite concentration was less than 1 μmol/L at pH levels less than 4, less than 10 μmol/L at pH levels 4 to 5, less than 100 μmol/L at pH levels greater than 5 to 6.99, and less than 200 μmol/L at pH levels of 7 or greater. As the reaction to form NDMA depends on the nitrite being protonated, Figure 1B shows the protonated nitrite concentration based on nitrite’s p*K*_a_, which is the negative log of the acid dissociation constant (*K*_a_) value. The lower the p*K*_a_, the stronger the acid. For nitrite, the p*K*_a_ value is 3.3, which means that at pH 3.3, 50% of nitrite will be protonated and 50% will be deprotonated. In Figure 1, with pH levels of less than 5, increases in total nitrite concentration are offset by the decreased amount of nitrite that is protonated, such that protonated nitrite remains approximately constant. With pH levels greater than 5, protonated nitrite concentration decreases rapidly as pH increases. eTable 1 and eFigure 1 in the Supplement summarize all studies with paired fasting nitrite and pH data by patient or study group.

### Association of Meals With Gastric Nitrite and pH

eTable 2 in the Supplement summarizes clinical studies that contain data on nonfasting gastric nitrite concentration and pH. Two studies^12,13^ reported the association of concentrated potassium nitrate solution boluses administered via nasogastric tube on gastric nitrite concentration. After nitrate is absorbed from the intestines, secretion into saliva occurs in a concentrated form that can then be converted to nitrite by nitrate-reducing bacteria on the back of the tongue. However, a bolus of nitrate solution is not reflective of physiology that occurs after consuming nitrate in food, as the meal will increase pH as well as the volume of food and liquids in the stomach, diluting the concentration of salivary nitrite that enters the stomach.

Regarding the effect of actual meals, 1 study^14,15^ from 45 years ago reported the effect of an extremely high-nitrite meal including canned luncheon meat from a time prior to modern regulations and technology that reduced nitrite in cured meats.^16^ Four other studies^17,18,19,20^ assessed gastric pH and nitrite every 30 or 60 minutes for 24 hours, thus including data from the fasting and fed state. Milton-Thompson et al^19^ reported median and upper range data from healthy volunteers before, during, and after cimetidine administration combined and binned by 1-unit pH increases (eFigure 2A in the Supplement).^19^ The upper range of nitrite concentration was approximately 1 μmol/L at pH levels less than 2, less than 20 μmol/L at pH levels less than 5, and less than 50 μmol/L at pH levels less than 7. Hall et al^17^ also reported data by 1-unit pH in patients with a history of Pólya partial gastrectomy or pernicious anemia as well as control participants (eFigure 2B in the Supplement).^17^ The 95th percentile nitrite concentration was less than 10 μmol/L at pH levels of less than 2, less than 100 μmol/L until pH levels of 5, and approximately 100 μmol/L at pH levels of 5 to 7. Note that Pólya partial gastrectomy resulted in significantly altered anatomy and physiology, predisposing patients to higher nitrite concentrations, similar to Billroth II partial gastrectomy.^21^ With both studies,^17,19^ the protonated nitrite concentration decreased substantially at pH levels greater than 4 (eFigure 2 in the Supplement). Overall, these studies provide a conservative upper bound for gastric fluid nitrite amounts at a pH level of less than 6 of approximately 100 μmol/L.

### Ranitidine in Simulated Gastric or Intestinal Fluid Studies

Because nitrite was unstable in the acidic conditions of simulated gastric fluid (eMethods in the Supplement), simulated gastric fluid solutions with added nitrite were prepared immediately prior to adding the ranitidine solution, and incubation was started immediately after mixing. A stock solution of sodium nitrite in water was prepared, and predetermined amounts of the nitrite stock solution were added to simulated gastric fluid solutions (100, 1000, 5000, and 10 000 μmol/L) or intestinal fluid solutions (10 000 μmol/L), while the concentration of ranitidine was kept constant by adding a 150-mg tablet. Experiments with 50 mL of simulated gastric fluid were performed across a range of pHs (1.2, 2.0, 3.0, and 5.0) with a supraphysiologic nitrite concentration (10 000 μmol/L). At the pH with maximal formation of NDMA (ie, 1.2), experiments were performed with decreasing nitrite concentrations (10 000, 5000, 1000, 100, and 0 μmol/L) in both 50 and 250 mL of simulated gastric fluid.

Ranitidine is highly water soluble, and the tablets were completely dissolved in 50 mL. Samples were incubated at 37 °C for 2 hours in a water bath shaker at 100 rpm based on the United States Pharmacopeia (USP <711>) 2-hour acid stage, simulating the time that a drug sample may stay in the stomach. Notably, the actual residence time of ranitidine in the fasted stomach may be much shorter (eg, water half-emptying time, 13 minutes^22^; half-life of drug solution, 4.2 minutes^23^). While food remains in the stomach longer during the digestive period, a drug dissolved in gastric fluid and/or liquids empties throughout the digestive period.^24^ After the incubation, 1-mL sample aliquots were taken, and 0.1 mL of 1 mol/L NaOH was added to prevent further potential NDMA formation (eMethods in the Supplement). Sample aliquots were kept at 4 °C before analysis for NDMA using the liquid chromatography with high-resolution mass spectrometer (LC-HRMS) method.

### LC-HRMS Method

An LC-HRMS analytical procedure was developed and validated for quantifying NDMA per regulatory guidelines. This is described in the eMethods in the Supplement.

### Statistical Analysis

NDMA data are presented as mean and SD of 3 independent samples run in duplicate. Data were collected and analyzed in Excel 2002 (Microsoft Corp).

## Results

**Figure 2.**
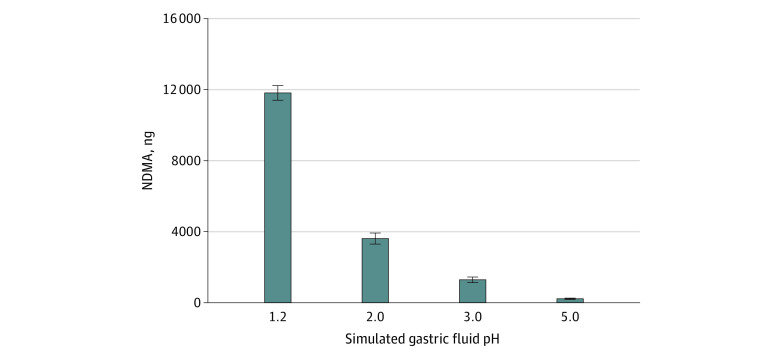
Formation of *N*-Nitrosodimethylamine (NDMA) From Ranitidine With a Supraphysiologic Nitrite Concentration With Increasing pH A plot of the amount of NDMA formed at different pHs in 50 mL of simulated gastric fluid with 3 mg/mL ranitidine and supraphysiologic 10 000 μmol/L nitrite after 2 hours incubation at 37 °C. The data represent the mean and SD of 3 independent samples run in duplicate, and the error bars show the SD of the measurement.

To test conditions under which ranitidine would be converted to NDMA, experiments were performed at a supraphysiologic nitrite concentration (10 000 μmol/L). At 10 000 μmol/L, the mean (SD) amount of NDMA detected in 50 mL simulated gastric fluid 2 hours after adding 1 ranitidine tablet (150 mg) decreased with increasing pH values of 1.2, 2.0, 3.0, and 5.0 from 11 822 (434) ng to 3616 (301) ng, 1310 (113) ng, and 222 (12) ng, respectively (Figure 2). Consistent with the pH dependence of the reaction, no NDMA was detected in 50 mL of simulated intestinal fluid (pH 6.8) with 10 000 μmol/L nitrite.

**Figure 3.**
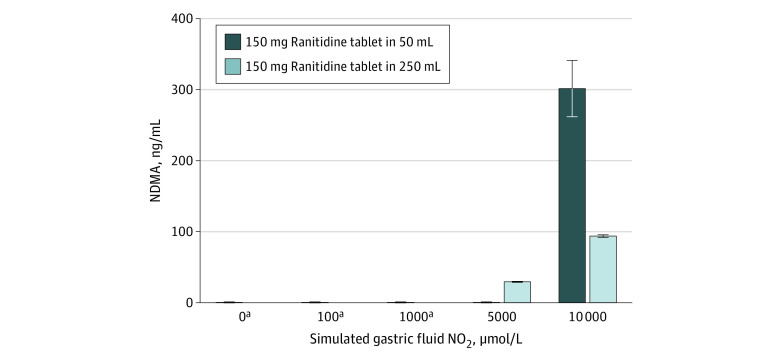
*N*-Nitrosodimethylamine (NDMA) Formation From Ranitidine at pH 1.2 in Simulated Gastric Fluid and Increasing Nitrite Concentration The amount of NDMA formed from a 150-mg ranitidine tablet with various nitrite concentrations under simulated gastric conditions in 50 mL or 250 mL simulated gastric fluid at a pH of 1.2. The values shown are the mean and SD of the 3 independent samples with 2 injections for each sample. ^a^The mean (SD) NDMA levels at 0, 100, 1000, and 5000 μmol/L for a 150-mg tablet in 50 mL simulated gastric fluid were 22 (2) ng, 21 (3) ng, 24 (2) ng, and 24 (3) ng, respectively, representing the background amount of NDMA present in the tablets used for the study within the error of the measurement. There was no detectable amount of NDMA at these levels (limit of detection, 0.33 ng/mL) when a 150-mg tablet was dissolved in 250 mL. Nitrite concentrations less than 100 μmol/L in simulated gastric fluid represent the range of NO_2_ amounts observed in clinical studies, with most concentrations being closer to 0 μmol/L than 100 μmol/L.

Subsequent experiments with varying nitrite concentrations (Figure 3) were performed in simulated gastric fluid at a pH of 1.2 because that pH was associated with the highest formation of NDMA when using 10 000 μmol/L nitrite. In 50 mL of simulated gastric fluid at pH 1.2 with no added nitrite, 22 (2) ng of NDMA was detected, which was the background NDMA amount present in the ranitidine tablets used for this study. Similarly, at the upper range of physiologic nitrite (100 μmol/L) and at nitrite concentrations as much as 50-fold greater (1000 or 5000 μmol/L), only background amounts of NDMA were observed (mean [SD], 21 [3] ng, 24 [2] ng, or 24 [3] ng, respectively). At 10 000 μmol/L nitrite, a mean (SD) of 15 067 (1983) ng of NDMA was detected in the 50 mL experiments.

With 250 mL of simulated gastric fluid, no NDMA was detected at the upper physiologic range of gastric nitrite concentration (100 μmol/L) or 10-fold physiologic (1000 μmol/L) nitrite concentrations, while additional NDMA was detected (mean [SD], 7353 [183] ng and 23 453 [443] ng) at 50- and 100-fold physiologic concentrations (5000 μmol/L and 10 000 μmol/L, respectively). Because the nitrite concentration was matched between the prior 50 mL simulated gastric fluid experiments and these experiments with 250 mL, the total amount of nitrite in simulated gastric fluid was 5 times greater in the 250 mL experiments. As 1 ranitidine tablet was added to both the 50 mL and 250 mL experiments, the ratio of nitrite to ranitidine molecules was 5 times greater in the 250 mL experiments compared with the 50 mL experiments.

## Discussion

**Figure 4.**
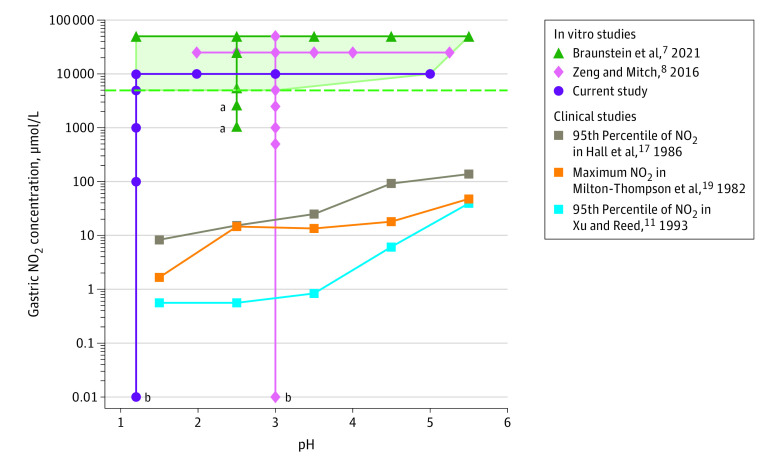
Nitrite (NO_2_) Concentration and pH in Ranitidine Simulated Gastric Fluid Studies Compared With Clinical Studies Lines connect the data points observed in the different clinical and nonclinical studies included in the plot. The light green shaded area represents the region where formation of NDMA was observed in simulated gastric fluid with supplemental nitrite across different pH values. This region is bounded at the bottom by a green dashed horizontal line, which indicates the lowest in vitro nitrite concentration (5000 μmol/L) with NDMA formation. Clinical nitrite concentrations from different clinical studies are plotted at pH midpoints (ie, results at pHs 2 to <3 are plotted at pH 2.5). Compared with the clinical nitrite concentration at pH levels of 1 to less than 2 (as tested in this study), the 5000 μmol/L value is approximately 600-folder greater than the 95th percentile clinical nitrite concentration values calculated from the Hall et al^17^ (8.2 μmol/L, combined fed and fasting), 3300-fold greater than the maximum clinical nitrite concentration measured by Milton-Thompson et al^19^ (1.5 μmol/L fasting), and 9000-fold greater than the 95th percentile clinical nitrite concentration calculated in the study by Xu and Reed^11^ (0.56 μmol/L, fasting). At the pH tested in Zeng and Mitch^8^ or Braunstein et al,^7^ the differences are approximately 350-fold (14.5 μmol/L, Hall et al^17^), 340-fold (14.9 μmol/L, Milton-Thompson et al^19^), and 8800-fold (0.57 μmol/L, Xu and Reed^11^). ^a^NDMA detected may be from background amounts in the ranitidine tablets used in the study by Braunstein et al.^7^ ^b^0 μmol/L.

The results of this in vitro study suggest that ranitidine does not convert to NDMA in the human stomach or small intestine under physiological conditions. At a pH of 1.2 with 50 mL simulated gastric fluid representative of the fasted stomach, ranitidine conversion to NDMA was not detected until the nitrite concentration was 10 000 μmol/L, which is approximately 18 000-fold greater than the clinically observed 95th percentile at pH levels less than 2 (Figure 1).^11^ When increasing the simulated gastric fluid volume to 250 mL, as may occur when consuming 200 mL of water, conversion of ranitidine to NDMA was detected when gastric nitrite was 5000 μmol/L instead of 10 000 μmol/L. However, this scenario is unlikely to be encountered in patients, as consuming water will increase pH and decrease the gastric nitrite concentration by dilution. Furthermore, 5000 µmol/L is approximately 600-fold greater than the 95th percentile of gastric nitrite at pH values of less than 2 from patients with 24-hour (combined fed and fasted) gastric fluid measurements (Figure 4 and eFigure 2 in the Supplement).^17^

As shown in Figure 4 and consistent with the results of this study, Zeng and Mitch^8^ also reported NDMA formation from ranitidine only at 5000 μmol/L nitrite and greater. In contrast, Braunstein et al^7^ (Figure 4) detected the same amount of NDMA at 1000 μmol/L nitrite and 2500 μmol/L nitrite, while at 5000 μmol/L nitrite the NDMA amount increased.^13^ Notably, Braunstein et al^7^ did not report the background amounts of NDMA present in the ranitidine product they used in the study. Thus, the constant amount of NDMA they detected at the lower nitrite concentrations (ie, 1000 μmol/L and 2500 μmol/L) may be the result of background NDMA being present in the ranitidine tablets before being placed in simulated gastric fluid and not the result of ranitidine-to-NDMA conversion in simulated gastric fluid. Zeng and Mitch have recently retracted or corrected reported NDMA findings in 2 journals^25,26^ because of analytical artifacts associated with the use of gas chromatography–mass spectrometry technology for measurements where ranitidine is present. The retraction and correction highlight the need for using a fit-for-purpose method for the analytical measurements in conjunction with conditions that as closely as practical reflect human physiology to assess the potential for drugs to be converted in vivo to nitrosamines. Regardless, the lowest nitrite concentration they studied (1000 μmol/L) was more than 1800-fold greater than the 95th percentile of fasting nitrite concentrations (Figure 1)^11^ and approximately 70-fold greater than the maximum range^19^ or 95th percentile^17^ of combined fed and fasting samples at pH levels between 2 and less than 3 (eFigure 2 in the Supplement). At other pH values, Braunstein et al^7^ used 50 000 μmol/L nitrite, which is approximately 88 000-fold greater than the 95th percentile of fasting nitrite at pH levels between 2 and less than 3 and approximately 3500-fold greater than combined fed and fasting (Figure 1 and eFigure 2 in the Supplement).^11,17,19^ None of the experiments in the study by Braunstein et al^7^ included physiological conditions.

As described in the Methods section and shown in eTable 2 in the Supplement, a study published in 1976 by Walters et al^14,15^ included canned luncheon meat with enough nitrite to bring the total meal’s sodium nitrite content to 24 mg (16 mg NO_2_^-^), which represents a worst-case scenario of nitrite intake from a past era. Since then, additional regulations in different countries were implemented to reduce the amount of nitrite in cured meat, and the industry evolved to reduce nitrite even further.^16,27,28,29,30^ In the United States, as of 2009, the mean daily exposure to nitrites from cured meats among those who eat cured meats was 0.25 mg per day, and the 90th percentile was 0.56 mg per day.^27,30^ Thus, the amount of nitrite in the meal described in Walters et al^14,15^ was 64 times higher than the mean and 29 times higher than the 90th percentile of cured meat eaters. Despite the high amount of nitrite in the meal in Walters et al,^14,15^ gastric nitrite only reached 290 μmol/L and occurred at pH 4.7, which is associated with a protonated nitrite concentration nearly identical to the fasting level.

Increased gastric nitrite concentrations have been proposed to be because of increased nitrate-to-nitrite reducing bacteria at higher pHs in the stomach. However, nitrite is known to be more stable at higher pH,^31^ and if more nitrite is present in gastric juice at a higher pH, then as pH decreases, nitrite will also decrease. Interestingly, when study participants in Mowat et al,^12^ who were receiving 40 mg of omeprazole for 4 weeks (fasting pH, 7.2), were administered 2 mmol of potassium nitrate by nasogastric tube, there was no increase in gastric nitrite immediately. Rather, nitrite reached the highest levels from 40 to 90 minutes after the potassium nitrate bolus.^12^ The lag time for elevated nitrite is consistent with the increase in gastric nitrite only occurring after the nitrate bolus was absorbed, secreted in concentrated form by salivary glands, converted to nitrite by nitrate-reducing bacteria in the oral cavity, and then nitrite entering the stomach with saliva.^32^

Questions regarding the potential for nitrosamines to form in vivo from drugs or food were asked approximately 50 years ago.^33^ As shown in this study and other work, because amine nitrosation reactions, which form nitrosamines, are very dependent on the chemical environment and the presence of a suitably reactive amine group, care should be taken in ascribing observed reactivity under nonphysiologic conditions as evidence for the potential of drugs such as ranitidine to form NDMA in humans. Like this study, use of available clinical data to assess test conditions that should be used for in vitro testing have been previously reported. For example, in 1985 Gillatt et al^10,34^ assessed the propensity of various amine-containing drugs to form nitrosamines with a very high nitrite concentration (40 000 μmol/L) and compared those results with what was determined as the upper bounds of simulated gastric nitrite concentration from available clinical data (25 μmol/L). Gillatt et al^10,34^ highlighted the importance of the nitrite concentration and noted that reactivity at the higher concentration was not always predictive of nitrosamine formation at the lower concentration. Furthermore, nonclinical mutagenicity studies with ranitidine from the early 1980s had used excessive nitrite concentrations, leading to questionable clinical relevance, as commented on by the authors of those studies.^35,36,37^

In this study, as well as in the study by Gillatt et al,^10,34^ the supraphysiologic nitrite concentrations used by in vitro studies^7,8^ were compared with nitrite concentrations observed in physiological gastric fluid clinical studies. In addition, the interplay of reactive nitrite and pH was evaluated. Overall, to investigate the potential for pharmaceuticals to lead to in vivo formation of NDMA or other nitrosamines, the test conditions used in vitro should include physiologically relevant conditions. When that was done with ranitidine in this study, in vitro formation of NDMA did not occur.

### Limitations

The in vitro model used in this study has inherent limitations and does not reflect all aspects of human physiology, but it does provide a method for assessing the potential for physiologic nitrite reactions with drugs in the gastric fluid to lead to NDMA formation. The in vitro studies performed here did not include gastric conditions in the presence of a meal aside from experiments at higher pH levels that occur following a meal, which led to decreased formation of NDMA. In addition, the lack of conversion of ranitidine to NDMA at simulated physiological conditions observed here was further supported by the accompanying randomized, placebo-controlled clinical trial,^38^ which demonstrated that ranitidine did not increase urinary excretion of NDMA across 2 different diets with, 1 containing large quantities of cured meats.

## Conclusions

In this in vitro study of ranitidine tablets added to simulated gastric fluid with different nitrite concentrations, ranitidine conversion to NDMA was not detected until nitrite was 5000 μmol/L, which is 50-fold greater than the upper range of physiologic gastric nitrite concentrations at acidic pH. These findings suggest that ranitidine is not converted to NDMA in gastric fluid at physiologic conditions and are further supported by the results of a recent clinical trial.^38^

## Notes

Supplement. **eMethods.** Supplementary Methods **eTable 1.** pH and Nitrite Concentration in Gastric Juice in Fasted Patients **eTable 2.** Effect of Food on Gastric Nitrite Concentration and pH **eFigure 1**. Clinical Studies Included in the Review of Gastric Nitrite Amounts **eFigure 2.** pH Dependence of Nitrite Concentration From the Clinical Data in Studies by Milton-Thompson et al and Hall et al **eFigure 3.** Effects of a Very Nitrite Rich Meal on Gastric Nitrite and pH After a Meal eReferences.

## References

[1] Zeldis JB, Friedman LS, Isselbacher KJ. Ranitidine: a new H2-receptor antagonist. N Engl J Med. 1983;309(22):1368-1373. doi:10.1056/NEJM198312013092206 6314139

[2] Kane SP. Ranitidine-drug use statistics, United States, 2008 to 2018. Accessed May 15, 2021. https://clincalc.com/DrugStats/Drugs/Ranitidine

[3] US Food and Drug Association. FDA updates and press announcements on NDMA in Zantac (ranitidine). April 16, 2020. Accessed June 6, 2020. https://www.fda.gov/drugs/drug-safety-and-availability/fda-updates-and-press-announcements-ndma-zantac-ranitidine

[4] World Health Organization. Some *N*-Nitroso Compounds. World Health Organization IARC; 1978.

[5] US Food and Drug Association. Control of nitrosamine impurities in human drugs: guidance for industry. September 2020. Accessed May 24, 2021. https://www.fda.gov/regulatory-information/search-fda-guidance-documents/control-nitrosamine-impurities-human-drugs

[6] King FJ, Searle AD, Urquhart MW. Ranitidine–investigations into the root cause for the presence of *N*-nitroso-*N*, *N*-dimethylamine in ranitidine hydrochloride drug substances and associated drug products. Organic Process Res Dev. 2020;24(12):2915−2926. doi:10.1021/acs.oprd.0c00462

[7] Braunstein LZ, Kantor ED, O’Connell K, . Analysis of ranitidine-associated *N*-nitrosodimethylamine production under simulated physiologic conditions. JAMA Netw Open. 2021;4(1):e2034766. doi:10.1001/jamanetworkopen.2020.34766 33512515 PMC7846938

[8] Zeng T, Mitch WA. Oral intake of ranitidine increases urinary excretion of *N*-nitrosodimethylamine. Carcinogenesis. 2016;37(6):625-634. doi:10.1093/carcin/bgw034 26992900

[9] Mirvish SS. Formation of *N*-nitroso compounds: chemistry, kinetics, and in vivo occurrence. Toxicol Appl Pharmacol. 1975;31(3):325-351. doi:10.1016/0041-008X(75)90255-0 238307

[10] Gillatt PN, Palmer RC, Smith PL, Walters CL, Reed PI. Susceptibilities of drugs to nitrosation under simulated gastric conditions. Food Chem Toxicol. 1985;23(9):849-855. doi:10.1016/0278-6915(85)90286-8 4043885

[11] Xu GP, Reed PI. *N*-nitroso compounds in fresh gastric juice and their relation to intragastric pH and nitrite employing an improved analytical method. Carcinogenesis. 1993;14(12):2547-2551. doi:10.1093/carcin/14.12.2547 8269625

[12] Mowat C, Carswell A, Wirz A, McColl KE. Omeprazole and dietary nitrate independently affect levels of vitamin C and nitrite in gastric juice. Gastroenterology. 1999;116(4):813-822. doi:10.1016/S0016-5085(99)70064-8 10092303

[13] Suzuki H, Iijima K, Moriya A, . Conditions for acid catalysed luminal nitrosation are maximal at the gastric cardia. Gut. 2003;52(8):1095-1101. doi:10.1136/gut.52.8.1095 12865265 PMC1773739

[14] Walters CL, Dyke CS, Saxby MJ. Nitrosation of food amines under stomach conditions. IARC Sci Publ. 1976;(14):181-193. 12086

[15] Walters CL, Carr FP, Dyke CS, Saxby MJ, Smith PL, Walker R. Nitrite sources and nitrosamine formation in vitro and in vivo. Food Cosmet Toxicol. 1979;17(5):473-479. doi:10.1016/0015-6264(79)90006-3 520980

[16] Keeton JT. History of nitrite and nitrate in food. In: Bryan NS, Loscalzo J, eds. Nitrite and Nitrate in Human Health and Disease. 2nd ed. Humana Press; 2016:85-97. doi:10.1007/978-1-60761-616-0_5

[17] Hall CN, Darkin D, Brimblecombe R, Cook AJ, Kirkham JS, Northfield TC. Evaluation of the nitrosamine hypothesis of gastric carcinogenesis in precancerous conditions. Gut. 1986;27(5):491-498. doi:10.1136/gut.27.5.491 3699560 PMC1433484

[18] Keighley MR, Youngs D, Poxon V, . Intragastric *N*-nitrosation is unlikely to be responsible for gastric carcinoma developing after operations for duodenal ulcer. Gut. 1984;25(3):238-245. doi:10.1136/gut.25.3.238 6698439 PMC1432290

[19] Milton-Thompson GJ, Lightfoot NF, Ahmet Z, . Intragastric acidity, bacteria, nitrite, and *N*-nitroso compounds before, during, and after cimetidine treatment. Lancet. 1982;1(8281):1091-1095. doi:10.1016/S0140-6736(82)92277-2 6122891

[20] Thomas JM, Misiewicz JJ, Cook AR, . Effects of one year’s treatment with ranitidine and of truncal vagotomy on gastric contents. Gut. 1987;28(6):726-738. doi:10.1136/gut.28.6.726 3623220 PMC1433039

[21] Schlag P, Böckler R, Ulrich H, Peter M, Merkle P, Herfarth C. Are nitrite and *N*-nitroso compounds in gastric juice risk factors for carcinoma in the operated stomach? Lancet. 1980;1(8171):727-729. doi:10.1016/S0140-6736(80)91229-5 6103154

[22] Mudie DM, Murray K, Hoad CL, . Quantification of gastrointestinal liquid volumes and distribution following a 240 mL dose of water in the fasted state. Mol Pharm. 2014;11(9):3039-3047. doi:10.1021/mp500210c 25115349

[23] Yamashita S, Kataoka M, Higashino H, . Measurement of drug concentration in the stomach after intragastric administration of drug solution to healthy volunteers: analysis of intragastric fluid dynamics and drug absorption. Pharm Res. 2013;30(4):951-958. doi:10.1007/s11095-012-0931-1 23179782

[24] Goyal RK, Guo Y, Mashimo H. Advances in the physiology of gastric emptying. Neurogastroenterol Motil. 2019;31(4):e13546. doi:10.1111/nmo.13546 30740834 PMC6850045

[25] Zeng T, Mitch WA. Retraction to: oral intake of ranitidine increases urinary excretion of *N*-nitrosodimethylamine. Carcinogenesis. Published online May 4, 2021. doi:10.1093/carcin/bgab029 26992900

[26] Zeng T, Mitch WA. Correction for "Contribution of N-Nitrosamines and Their Precursors to Domestic Sewage by Greywaters and Blackwaters". Environ Sci Technol. 2021;55(8):5602. 33761234 10.1021/acs.est.1c01757

[27] Lee HS. Exposure estimates of nitrite and nitrate from consumption of cured meat products by the US population. Food Addit Contam Part A Chem Anal Control Expo Risk Assess. 2018;35(1):29-39. doi:10.1080/19440049.2017.1400696 29095117

[28] Cassens RG. Use of sodium nitrite in cured meats today. Food Technol. 1995;49:72–80.

[29] National Research Council. The Health Effects of Nitrate, Nitrite, and *N*-Nitroso Compounds: Part 1 of a 2-Part Study. The National Academies Press; 1981.

[30] Nuñez De González MT, Osburn WN, Hardin MD, . Survey of residual nitrite and nitrate in conventional and organic/natural/uncured/indirectly cured meats available at retail in the United States. J Agric Food Chem. 2012;60(15):3981-3990. doi:10.1021/jf204611k 22414374

[31] López-Rodríguez R, McManus JA, Murphy NS, Ott MA, Burns MJ. Pathways for *N*-nitroso compound formation: secondary amines and beyond. Organic Process Res Dev. 2020;24(9):1558-1585. doi:10.1021/acs.oprd.0c00323

[32] Mowat C, McColl KE. Alterations in intragastric nitrite and vitamin C levels during acid inhibitory therapy. Best Pract Res Clin Gastroenterol. 2001;15(3):523-537. doi:10.1053/bega.2000.0196 11403544

[33] Lijinsky W, Conrad E, Van de Bogart R. Carcinogenic nitrosamines formed by drug-nitrite interactions. Nature. 1972;239(5368):165-167. doi:10.1038/239165b0 4561965

[34] Gillatt PN, Hart RJ, Walters CL, Reed PI. Susceptibilities of drugs to nitrosation under standardized chemical conditions. Food Chem Toxicol. 1984;22(4):269-274. doi:10.1016/0278-6915(84)90005-X 6539274

[35] Brambilla G, Cavanna M, Faggin P, . Genotoxic effects in rodents given high oral doses of ranitidine and sodium nitrite. Carcinogenesis. 1983;4(10):1281-1285. doi:10.1093/carcin/4.10.1281 6311451

[36] Martelli A, Fugassa E, Voci A, Brambilla G. Unscheduled DNA synthesis induced by nitrosated ranitidine in primary cultures of rat hepatocytes. Mutat Res. 1983;122(3-4):373-376. doi:10.1016/0165-7992(83)90022-2 6318101

[37] Maura A, Pino A, Robbiano L, . DNA damage induced by nitrosated ranitidine in cultured mammalian cells. Toxicol Lett. 1983;18(1-2):97-102. doi:10.1016/0378-4274(83)90077-2 6312641

[38] Florian J, Matta M, DePalma R, . Effect of oral ranitidine administration on urinary excretion of *N*-nitrosodimethylamine (NDMA): a randomized clinical trial. JAMA. Published online June 28, 2021. doi:10.1001/jama.2021.9199 PMC8240005 34180947

