# Errors in Figure Captions

**DOI:** 10.1001/jamanetworkopen.2021.23189

**Published:** 2021-07-20

In the Original Investigation “In Vitro Analysis of *N*-Nitrosodimethylamine (NDMA) Formation From Ranitidine Under Simulated Gastrointestinal Conditions,”^1^ published June 28, 2021, there were errors in the captions for Figures 2, 3, and 4 and eFigure 3. The level of simulated gastric fluid in Figure 2 should have read 150 mg. The statement in Figure 3 describing the nondetectable level of NDMA when a 150-mg tablet was dissolved in 250 mL should have noted that this result was not observed at 5000 and 10 000 μmol/L. In Figure 4, the maximum clinical nitrite concentration in the article by Milton-Thompson et al should have read 1.5 μmol/L, combined fed and fasting. This article has been corrected.^1^

## References

[1] Gao Z, Karfunkle M, Ye W, . In vitro analysis of *N*-nitrosodimethylamine (NDMA) formation from ranitidine under simulated gastrointestinal conditions. JAMA Netw Open. 2021;4(6):e2118253. doi:10.1001/jamanetworkopen.2021.18253 34181009 PMC8239951

