# Supplementary material for: In Vitro Analysis of N-Nitrosodimethylamine (NDMA) Formation From Ranitidine Under Simulated Gastrointestinal Conditions
Source: JAMA Netw Open. 2021 Jun 28;4(6):e2118253. doi: 10.1001/jamanetworkopen.2021.18253 (PMC8239951; doi:10.1001/jamanetworkopen.2021.18253)
Supplement: Supplement. — eMethods. Supplementary Methods eTable 1. pH and Nitrite Concentration in Gastric Juice in Fasted Patients eTable 2. Effect of Food on Gastric Nitrite Concentration and pH eFigure 1. Clinical Studies Included in the Review of Gastric Nitrite Amounts eFigure 2. pH Dependence of Nitrite Concentration From the Clinical Data in Studies by Milton-Thompson et al and Hall et al eFigure 3. Effects of a Very Nitrite Rich Meal on Gastric Nitrite and pH After a Meal eReferences. [file jamanetwopen-e2118253-s001.pdf]

Supplemental Online Content

Gao Z, Karfunkle M, Ye W, et al. In vitro analysis of *N*-nitrosodimethylamine (NDMA) formation from ranitidine under simulated gastrointestinal conditions. *JAMA Netw Open.* 2021;4(6):e2118253. doi:10.1001/jamanetworkopen.2021.18253

eMethods. Supplementary Methods

eTable 1. pH and Nitrite Concentration in Gastric Juice in Fasted Patients

eTable 2. Effect of Food on Gastric Nitrite Concentration and pH

eFigure 1. Clinical Studies Included in the Review of Gastric Nitrite Amounts

eFigure 2. pH Dependence of Nitrite Concentration From the Clinical Data in Studies by Milton-Thompson et al and Hall et al

eFigure 3. Effects of a Very Nitrite Rich Meal on Gastric Nitrite and pH After a Meal

eReferences.

This supplemental material has been provided by the authors to give readers additional information about their work.

eMethods. Supplementary Methods

For this study, International Council for Harmonisation (ICH Q2(R1)) validation principles were applied to establish a fit-for purpose analytical procedure for measurement of NDMA in simulated gastric fluid. In the development of the method key steps were necessary to assure the quality of the data (*e.g.,* measurement of the background amount of NDMA in the tablets used for the study, mixing ranitidine with SGF nitrite solutions prepared immediately before the start of the reaction and quenching with base to stop further reaction).

<u>Materials:</u>

In the simulated GI studies, the following materials were used: Sodium chloride from Fisher Chemical, catalog number S271-3; pepsin from Sigma-Aldrich, catalog number P1725-500G; sodium nitrite from Alfa Aesar, catalog number 14244; hydrochloric acid from Fisher Chemical, catalog number 320331-2.5L; sodium hydroxide from Sigma-Aldrich, catalog number 72064-500ML; ranitidine HCl from TCI Chemical, catalog number R0073; and Zantac (ranitidine) 150 mg tablets, manufactured by Sanofi, NDC number 41167-0310-6. In the anion exchange chromatography, the following materials were used: Nitrite ion chromatography reference standard from SPEX CertiPrep, catalog number ASNO29-2Y; hydroxide from Sigma-Aldrich, catalog number 72064-500ML; and Optima™ LC/MS grade purified water from Fisher Chemical. In the LC-HRMS analysis, the following LC/MS grade materials, all sourced from Fisher Chemical, were used: Formic acid, catalog number A117-50; Acetonitrile, catalog number A955-4; methanol, catalog number A456-4; and Optima™ LC/MS grade purified water.

<u>Ranitidine tablet assay:</u>

150 mg ranitidine tablets were tested in this study. Assay for ranitidine products was performed according to the USP monograph for ranitidine tablets (USP43-NF38-3847). Ten tablets were placed in a flask containing 250 mL mobile phase (85% MeOH and 15% 0.1 M aqueous ammonium acetate). The USP tablets were 100.3% of the label claim, meeting the USP specification.

<u>Liquid chromatography with high resolution mass spectrometer (LC-HRMS) method:</u>

NDMA was separated from ranitidine by reverse phase chromatography and was detected by a high-resolution and high-mass accuracy mass spectrometer. A ng/mL detection limit was achieved by monitoring the accurate m/z value of the protonated impurity ion. The limit of detection (LOD) and limit of quantitation (LOQ) were 0.33 and 1.0 ng/mL, respectively. Quantitation was performed by comparing the peak area of the NDMA extracted ion chromatogram from the samples to that from external reference standard.

<u>Preparation of simulated gastric fluid and simulated intestinal fluid:</u>

Simulated Gastric Fluid (SGF) was prepared following United States Pharmacopoeia (USP), Reagents and Reference Tables, Solutions (USP42-NF37 – 6168). The USP method calls for 3.2 g of pepsin with enzyme activity between 800 and 2500, per liter of SGF. The pepsin used in this study had activity of 400, so 6.4 g of this pepsin was used per liter of SGF. Simulated Intestinal Fluid (SIF) was prepared following USP, Reagents and Reference Tables, Solutions (USP42-NF37 – 6169). Pancreatin was not used.

<u>Ion chromatography method to assess sodium nitrite solution stability:</u>

An anion exchange chromatography (Thermo Scientific™ Dionex™ ICS-5000 IC system) method was developed and validated following ICH Q2(R1) for the detection and quantitation of nitrite in ranitidine drug substance and drug product. The limit of detection (LOD), limit of quantitation (LOQ) and linear range of the method are 0.025, 0.100 and 0.1 – 100 µg/mL respectively. To compare the stability of nitrite in SGF and water, solutions with 1 mM sodium nitrite in SGF and purified water were prepared at room temperature. Each nitrite solution was transferred to HPLC vials and analyzed repeatedly over a 19-hour period at room temperature. One replicate was prepared for water and one for SGF. Alternating injections were taken starting with the sample prepared in SGF.

<u>Nitrite stability in SGF:</u>

© 2021 Gao Z et al. *JAMA Network Open.*

Nitrite is reported to be unstable in acidic solutions, and therefore for method development purposes the stability of sodium nitrite in SGF or water was determined. The data showed that nitrite concentrations remained constant when prepared in purified water, while in SGF at pH 1.2 about 60% of the nitrite was lost over a 19-hour period. The results agree with previous reports that sodium nitrite is unstable in acidic conditions.[1,2]

Need for quenching with base to minimize variability in NDMA measurements in vitro:

In initial method development studies, inconsistent NDMA concentrations were observed in individual samples. The inconsistent NDMA concentrations were hypothesized to occur because continuous nitrosation could occur during the time period after sampling but before sample analysis. Previous studies recommended quenching the reaction with sodium hydroxide to stop nitrosation after taking sample aliquots.[3] Thus, the impact of quenching sample aliquots was investigated.

The effect of quenching with 1 M NaOH solution under 1:1 aliquot/NaOH (v/v) ratio on NDMA was evaluated to reduced observed variability in NDMA values. Two injections were performed for each sample, with a time interval of five hours between the two injections. The results indicated over a 25% difference between observed NDMA without quenching of the sample aliquot, and less than 5% difference observed with sample quenching. Therefore, quenching was performed on all subsequent samples.

Boundary conditions for physiologic nitrite concentrations in gastric juice

To investigate the physiologic nitrite concentrations in gastric juice. Two independent reviewers extracted data on nitrite concentrations and pH in gastric juice, as well as on the context and characteristics of proton pump inhibitors (PPIs) and histamine-2 (H2) blocker intervention and assessed risk of bias. Twenty-six studies were included in the review; eFigure 1, eTable 1 and eTable 2 provide details from these clinical studies.

© 2021 Gao Z et al. *JAMA Network Open.*

eTable 1. pH and Nitrite Concentration in Gastric Juice from Fasted Patients

Group	Subgroup	Study	No. of Study Participants	pH	Nitrite (µmol/l)
Healthy	Healthy volunteers	Morris et al., 1988[4]	8	2.4 (0.5)[c]	4.2 (4.8)[c]
		Suzuki et al., 2003[5]	17	1.9[b]◊	0.4 (0.3)[c]
		Verdu et al., 1994[6]	14	1.9[a]	NR
		Vermeer et al., 2001[7]	13	2.5 (1.6)[c]	15 (7)[c]
		Walters et al., 1979[8]	6	2.1[b]	9.33 (5.45)[d]
		Watt et al., 1984b[9]	23	1.57[a]	0[a]
Patients	Patients undergoing endoscopy with no abnormal gastric findings	De Bernardinis et al., 1983[10]	15	1.7[b]	2.5[b]
		Guadagni et al., 1996[11]	20	2.04 (0.78)[c]	1.67 (2.05)[c]
		Ruddell et al., 1976[12]	18	1.4[b]□	1.7 (0.5)[d]
		Ruddell et al., 1978[13]	13	1.2-2.8[e]	2.7 (0.6)[d]
		Sobala et al., 1991[14]	12	2[a]	10.5[a]
		Schlag et al., 1980[15]	26	2.1[b]	5.7[b]
		Schlag et al., 1982[16]	26	2.35[b]	0.72[b]
		Ruddell et al., 1976[12] (Hypochorhydric normal)	5	2.7[b]□	25.6 (3.6)[d]
	Duodenal ulcer	De Bernardinis et al., 1983[10]	10	1.5[b]	2.5[b]
		Ruddell et al., 1976[12]	21	1.4[b]□	1.3 (0.3)[d]
		Schlag et al., 1982[16]	20	2.07[b]	0.76[b]
		Stockbrugger et al., 1982[17]	14	1.57 (0.09)[d]	1.6 (0.7)[d]
	Gastric ulcer	De Bernardinis et al., 1983[10] (Type I Johnson)	4	7.2[b]	23.3[b]
		De Bernardinis et al., 1983[10] (Type II and III Johnson)	6	1.8[b]	5.6[b]
		Ruddell et al., 1976[12]	12	1.7[b]□	7.9 (4.1)[d]
		Stockbrugger et al., 1982[17]	9	2.07 (0.55)[d]	5.9 (4.7)[d]
		Schlag et al., 1982[16]	11	2.07[b]	0.77[b]
	Chronic superficial gastritis	De Bernardinis et al., 1983[10]	5	4.8[b]	11.9[b]
		Schlag et al., 1982[16] (Superficial) (Moderate) (Severe)	10<br>12<br>10	2.44[b]<br>2.12[b]<br>2.21[b]	0.72[b]<br>1[b]<br>2.12[b]

© 2021 Gao Z et al. *JAMA Network Open.*

Group	Subgroup	Study	No. of Study Participants	pH	Nitrite (µmol/l)
Patients	Chronic superficial gastritis	Sobala et al., 1991[14]<br>(Chronic superficial gastritis)<br>(Reflux gastritis)	 18<br>9	 2.1[a]<br>2.4[a]	 10.9[a]<br>16.8[a]
		Tari et al., 2003[18]	22	6.17[a]	39.33[a]
	Chronic atrophic gastritis	De Bernardinis et al., 1983[10]	4	7.0[b]	28.3[b]
		Sobala et al., 1991[14]	17	3.1[a]	17.2[a]
		Schlag et al., 1982[16]	19	4.0[b]	16.95[b]
	Pernicious anemia	Ruddell et al., 1978[13]	13	7<br>("Neutral")	120 (20)[d]
	Gastric cancer	De Bernardinis et al., 1983[10]<br>(Undifferentiated carcinoma)<br>(Adenocarcinoma)	 2<br>5	 7.0[b]<br>6.8[b]	 26.0[b]<br>24.5[b]
		Guadagni et al., 1996[11]<br>(Adenocarcinoma)	14	7.31<br>(0.74)[c]	33.93<br>(16.64)[c]
		Ruddell et al., 1976[12]	6	2.3[b]□	38.81<br>(14.7)[d]
		Tari et al., 2003[18]	18	7.3[a]	128.1[a]
Operated stomach	Endoscopic mucosal resection	Tari et al., 2003[18]	16	7.1[a]	108.49[b]
	Roux-Y	Morris et al., 1988[4]	6	5.2 (10.8)[c]	14 (4.3)[c]
	Billroth I	De Bernardinis et al., 1983[10]	8	5.8[b]	17.6[b]
		Schlag et al., 1980[15]	10	4.5[b]	33.7[b]
	Billroth II	De Bernardinis et al., 1983[10]	12	7.41[b]	35.1[b]
		Morris et al., 1988[4]	5	5.3 (0.7)[c]	6.3 (1.8)[c]
		Schlag et al., 1980[15]	22	6.5[b]	579[b]
	Partial gastrostomies	Guadagni et al. 1996[11]	45	6.47<br>(1.12)[c]	20.27<br>(15.57)[c]
	Proximal-gastral vagotomy	Schlag et al., 1980[15]	12	2.2[b]	8.5[b]
	Vagotomy and gastrojejunostomy patients	Watt et al., 1984b[9]	51	5.18[a]	13.1[a]
	Vagotomy	Krytopoulos et al., 1985[19]<br>Time postoperative:<br>8-10 days<br>1-12 months	 <br>12<br>6	 <br>2.4[b]<br>4.4[b]	 <br>3.4[b]<br>9.6[b]
	Gastrectomy	Krytopoulos et al., 1985[19]<br>Time postoperative:<br>8-10 days<br>1-12 months	 <br>15<br>7	 <br>4.8[b]<br>6.8[b]	 <br>3.5[b]<br>27.9[b]

© 2021 Gao Z et al. *JAMA Network Open.*

Group	Subgroup	Study	No. of Study Participants	pH	Nitrite (µmol/l)
Operated stomach	Included Vagotomy, gastroenterostomy Billroth I, Billroth II and Gastroenterostomy	Watt et al., 1984a[20]		NR	
		Chronic superficial gastritis	35		3.9[a]
		Atrophic gastritis/intestinal metaplasia	30		13.1
		Mild dysplasia	21		13.0
		Moderate/severe dysplasia	13		90.1
		Carcinoma	1		173.0
Mixed population	Included superficial gastritis, chronic atrophic gastritis, duodenal ulcer, gastric ulcer, gastric carcinoma, reflux esophagitis, pernicious anemia as well as normal gastric histology, and also, those with vagotomy or partial gastrectomy.	Dang Vu et al., 1994[21]	146 ∆		
		All study participants divided into 1-unit increment pH groups.		1.0 – 1.99	0.50[b]
				2.0 – 2.99	0.44
				3.0 – 3.99	0.91
				4.0 – 4.99	2.44
				5.0 – 5.99	20.91
				6..0 – 6.99	22.52
				7.0 – 7.99	27.67
				>8.0	19.14
		Thorens et al., 1996[22]	47#		
		Before treatment			
		Cimetidine	NR	NR	28.6 (9.1)[d]
		Omeprazole	NR	NR	81.4 (51.2)
		After treatment			
		Cimetidine	18	2.0 (0.2)[d]	44 (15.9)
		Omeprazole	19	4.2 (0.5)	34.6 (11.3)
		Krytopoulos et al. 1985[19]	56		
		Before vagotomy		2.2[b]	2.8[b]
		Before gastrectomy		3.45[b]	10.3[b]
		Pignatelli et al., 1993[23]			
		United Kingdom	41	≤4	13.0 (1.3)[d]
		United Kingdom	10	>4	36.7 (7.3)
		France	51	≤4	21.4 (1.6)
		France	25	>4	48.0 (7.0)
		Colombia	24	≤4	20.5 (3.8)
		Colombia	28	>4	78.1 (18.0)

© 2021 Gao Z et al. *JAMA Network Open.*

Group	Subgroup	Study	No. of Study Participants	pH	Nitrite (μmol/l)
Mixed population	Included superficial gastritis, chronic atrophic gastritis, duodenal ulcer, gastric ulcer, gastric carcinoma, reflux esophagitis, pernicious anemia as well as normal gastric histology, and also, those with vagotomy or partial gastrectomy.	Xu et al., 1993[24] All study participants' samples divided into 1-unit increment pH groups.	176	1.13-1.99	0.12 (0.02)[d]
			66	2.00-2.99	0.17 (0.03)
			26	3.00-3.99	0.33 (0.06)
			18	4.00-4.99	2.55 (0.53)
			17	5.00-5.99	8.75 (4.68)
			39	6.00-6.99	25.64 (5.78)
			100	7.00-7.99	53.59 (8.50)
			15	8.00-8.42	48.49 (7.53)

Abbreviations: No., Number; NR, Not Reported.
[a] Median.
[b] Mean.
[c] Mean (Standard Deviation).
[d] Mean (Standard Error of the Mean).
[e] Range.
Ten of 47 patients were excluded during the study.
Δ All patients or samples were grouped together and divided into different pH groups.
□ Convert hydrogen ion concentration to pH.
◊ Measure nitrite and pH in proximal stomach.

© 2021 Gao Z et al. *JAMA Network Open.*

eTable2. Effect of Food on Gastric Nitrite Concentration and pH

Study	Population	Group	No. of Study Samples	pH (mean or median)	Nitrite (µmol/l) (mean or median)
**Effect of concentrated potassium nitrate solution boluses administered via nasogastric tube**					
Mowat et al., 1999[2]	Healthy volunteer participants who were administered 2 mmol potassium nitrate.	Fasted, no omeprazole<br>Nitrite meal, no omeprazole<br>Fasted, omeprazole<br>Nitrite meal, omeprazole	20<br>20<br>20<br>20	1.4[a]<br>1.4<br>7.2<br>7.2	0[a]<br>0<br>13<br>154
Suzuki et al., 2003[5]	Healthy volunteer participants who were administered 2 mmol potassium nitrate.	Fasted<br>Nitrite meal	17<br>17	1.9[b]◊<br>1.7	0.4[b]<br>8.9
**Effect or an extremely high nitrite meal**					
Walters et al., 1976[25]<br>Walters et al., 1979[8]	The effect of dietary nitrite on gastric nitrite levels in six volunteers who consumed a meal with an extremely high amount of nitrite from canned luncheon meat.	Fasted<br>Nitrite meal (40 min)	6<br>6	2.1[b]<br>4.7	9.33[b]<br>290
**24-hour gastric aspiration studies**					
Hall et al., 1986[26]	A 24-hour gastric aspiration study was carried out on 9 Pólya gastrectomy, 8 pernicious anemia, and 9 matched control subjects. The pH and nitrite were assessed every 30 minutes during the day and every 60 minutes at night.	All study participants' samples divided into 1-unit increment pH groups.	55*<br>33<br>26<br>27<br>111<br>132<br>13	1.15 – 1.99<br>2.0 – 2.99<br>3.0 – 3.99<br>4.0 – 4.99<br>5.0 – 5.99<br>6.0 – 6.99<br>7.0 – 7.92	1.67[b]<br>3.85<br>7.38<br>35.36<br>45.48<br>39.46<br>22.77
Keighley et al., 1984[27]	Hourly median values for pH and nitrite data over 24 hours in 24 patients after duodenal ulcer surgery and 8 controls without surgery.	Controls<br>Proximal gastric vagotomy<br>Truncal vagotomy<br>Vagotomy combined with antrectomy	8<br>7<br>7<br>8	<5[c]<br><6<br><6.2<br><6.1	<25[c]<br><25<br><25<br><30
Milton-Thompson et al., 1982[1]	Multiple samples were obtained from 8 healthy volunteers before, during and after cimetidine treatment. The pH and nitrite were assessed every 30 minutes during the day and every 60 minutes at night.	All study participants' samples divided into 1-unit increment pH groups.	56*<br>456<br>136<br>80<br>55<br>24<br>26	1.0 – 1.99<br>2.0 – 2.99<br>3.0 – 3.99<br>4.0 – 4.99<br>5.0 – 5.99<br>6.0 – 6.99<br>7.0 – 7.99	0[a]<br>0<br>0<br>0<br>2.5<br>6<br>7
Thomas et al., 1987[28]	15 patients with peptic ulcer who underwent 24-hour assessments, before ranitidine treatment, upon completing 6-weeks treatment with oral ranitidine 150 mg twice a day, on maintenance 150 mg daily for 9 to 12 months and 1 month after stopping the drug.	Before treatment<br>Week 6<br>Month 3<br>Month 9-12<br>Month 13	15<br>15<br>13<br>12<br>10	1.7[a]<br>3.0<br>2.0<br>2.0<br>1.8	<1.3[a]<br>2.9<br>4.2<br>5.1<br>1.5

Abbreviation: No., Number

[a] Median

[b] Mean

[c] Maximum median value for nitrite or pH during the day

* Samples

◊ Measure nitrite and pH in proximal stomach.

© 2021 Gao Z et al. *JAMA Network Open.*

eFigure 1. Clinical Studies Included in the Review of Gastric Nitrite Amounts

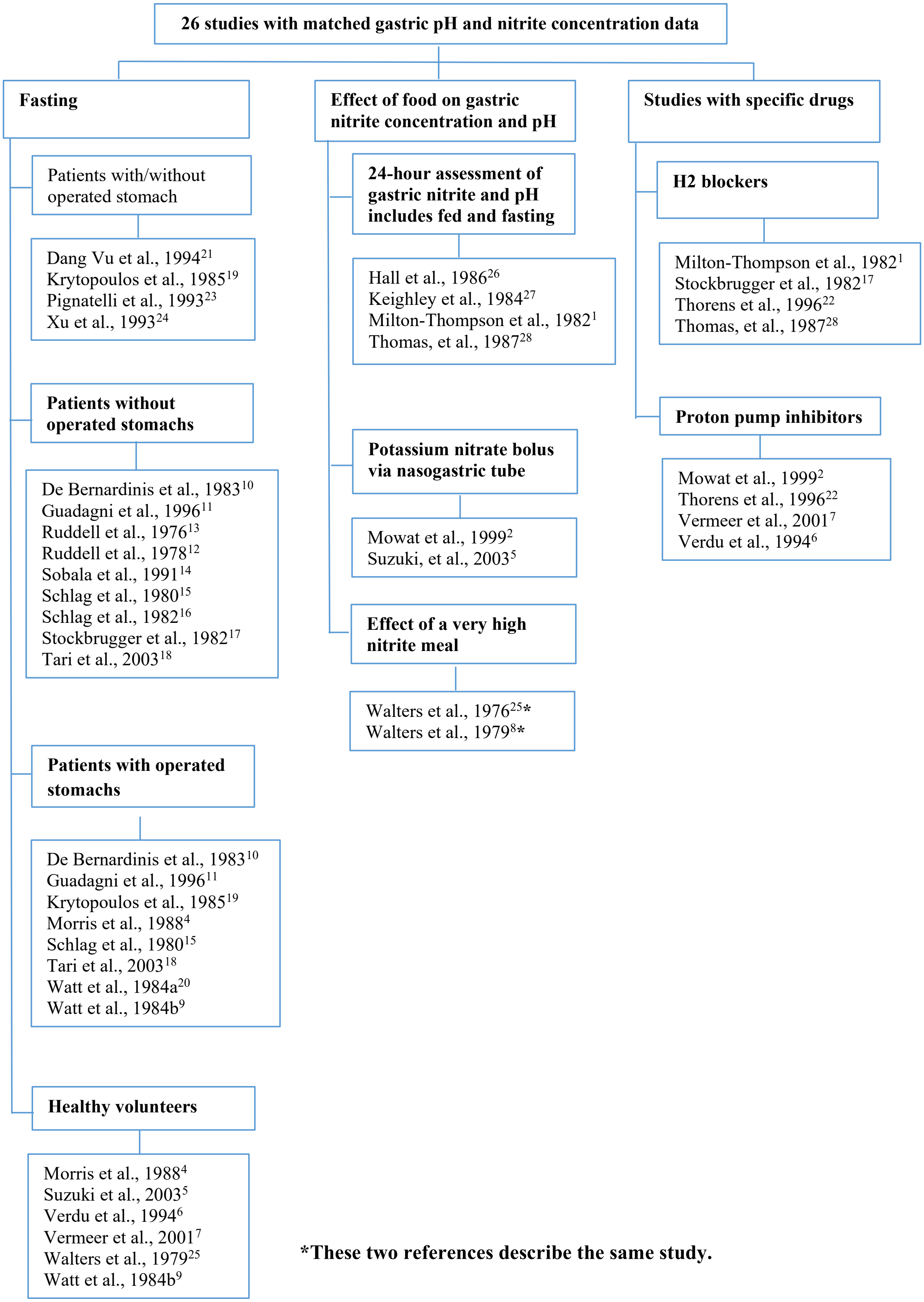

***These two references describe the same study.**

© 2021 Gao Z et al. *JAMA Network Open.*

eFigure 2. pH Dependence of Nitrite Concentration From the Clinical Data in Studies by Milton-Thompson et al and Hall et al

Panel A: Milton-Thompson et al.– 24 h Combined Fed and Fasted with Cimetidine

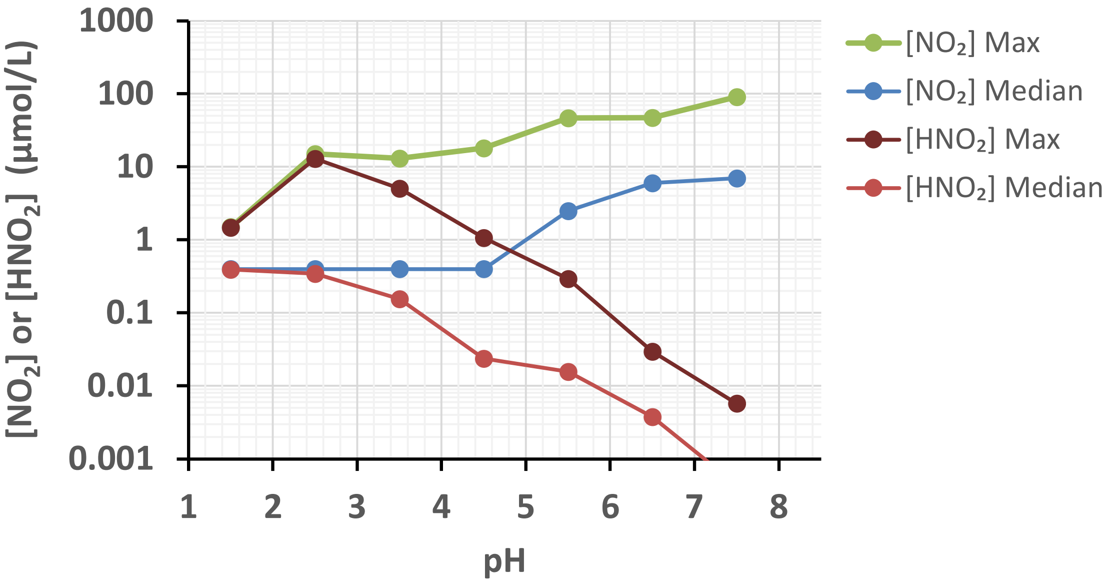

Panel B: Hall et al. – 24 h Pólya Gastrectomy, Pernicious Anemia and Control

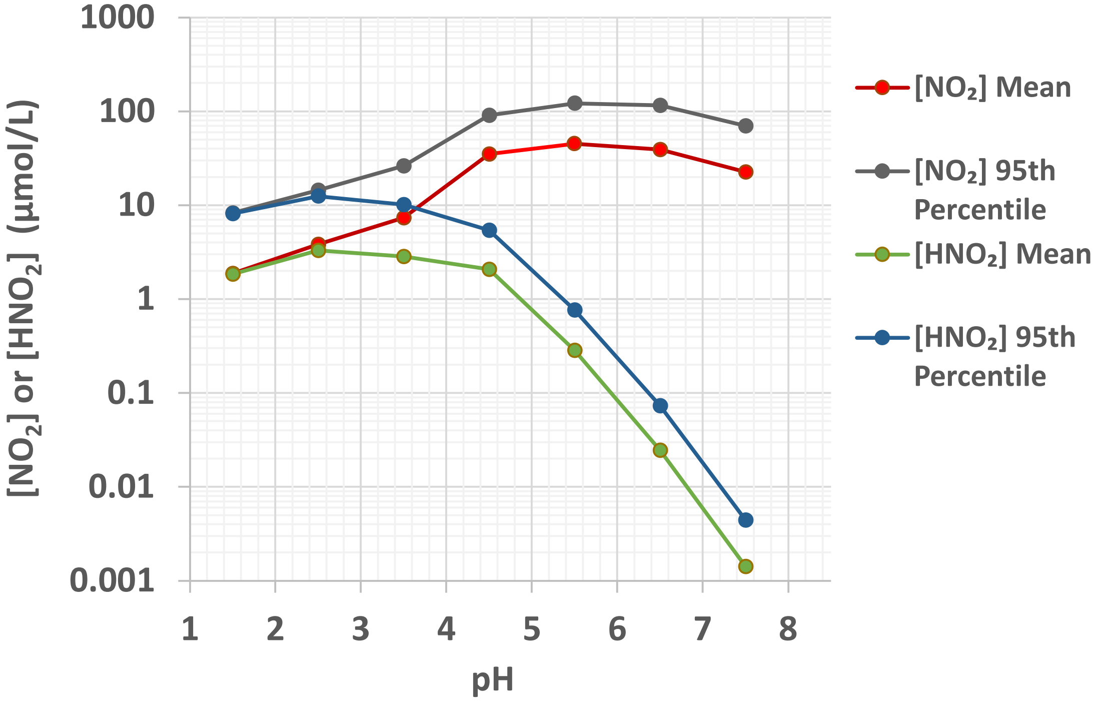

eFigure 2: Plots of the nitrite concentration ([NO_2] µmol/L) or calculated protonated nitrite ([HNO_2]) in gastric fluid from Hall *et al.*,[26] or Milton-Thompson *et al.*[1] clinical studies. Milton-Thompson *et al.,* reported median and upper range data from healthy volunteers before, during and after cimetidine administration combined and binned by 1-unit pH increases (mid-point pH is plotted for each 1 pH unit group). In **Panel A**, data from Milton-Thompson *et al.,*

© 2021 Gao Z et al. *JAMA Network Open.*

are shown that depict the median nitrite concentration (in blue) and max values (in green). In addition, the median (in red) and the maximum (dark red) calculated concentration of protonated nitrite ([HNO_2]) at each pH (based on the nitrite pKa of 3.3) are plotted. Hall *et al.,* also reported data by 1-unit pH groups in patients with a history of Pólya gastrectomy or pernicious anemia, as well as controls. In **Panel B, data** from Hall *et al.,* data are shown that depict the mean nitrite concentration ([NO_2] in red) versus pH (mid-point pH is plotted from 1 unit pH groups) and the calculated 95th percentile of nitrite concentration ([NO_2] 95th percentile in grey) based on the standard deviation. The concentration of protonated nitrite present ([HNO_2] mean in green) at each pH (based on the nitrite pKa of 3.3) is plotted along with the accompanying 95th percentile values ([HNO_2] 95th percentile in dark blue).

eFigure 3: Effects of a Very Nitrite Rich Meal on Gastric Nitrite and pH After a Meal

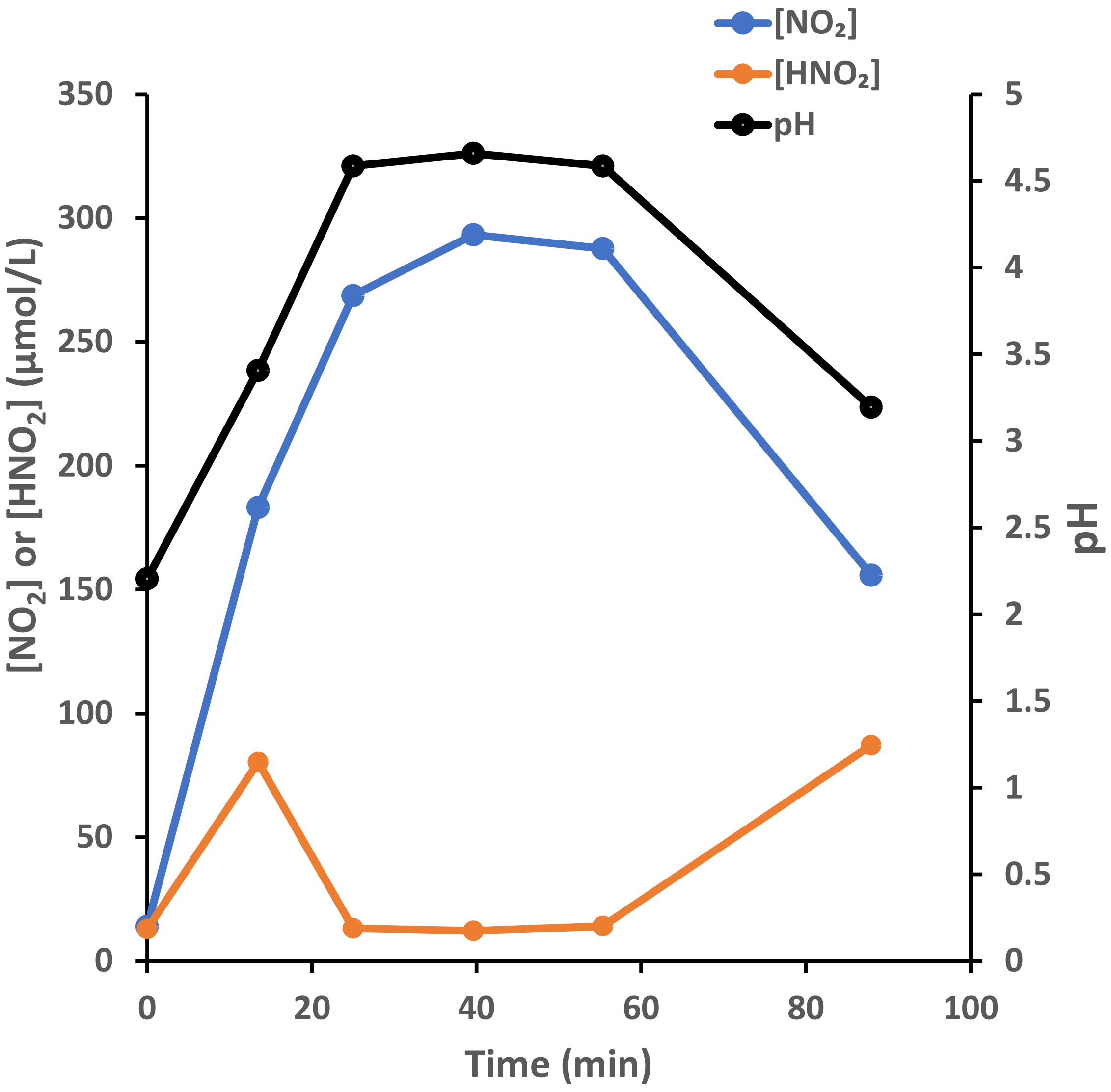

eFigure 3: A plot of human gastric nitrite concentration [NO_2] (or calculated protonated nitrite [HNO_2]) or pH versus time after ingesting a meal with a very high nitrite content (described in main text). With ingestion of the meal, the pH and the nitrite concentrations increase to 4.7 and 290 µmol/L, respectively. Importantly for potential nitrosation reactions, the percentage of nitrite that is protonated decreases substantially with increasing pH such that when nitrite concentration is highest after the meal, the protonated nitrite concentration [HNO_2] is approximately the same as fasting levels before the mean. Protonated nitrite is the reactive species necessary for nitrosamines to form.

© 2021 Gao Z et al. *JAMA Network Open.*

eReferences

1. Milton-Thompson GJ, Lightfoot NF, Ahmet Z, et al. Intragastric acidity, bacteria, nitrite, and N-nitroso compounds before, during, and after cimetidine treatment. *Lancet.* 1982;1(8281):1091-1095.

2. Mowat C, Carswell A, Wirz A, McColl KE. Omeprazole and dietary nitrate independently affect levels of vitamin C and nitrite in gastric juice. *Gastroenterology.* 1999;116(4):813-822.

3. Zeng T, Mitch WA. Oral intake of ranitidine increases urinary excretion of N-nitrosodimethylamine. *Carcinogenesis.* 2016;37(6):625-634.

4. Morris DL, Poxon V, Youngs D, Keighley M. Gastric juice factors after Roux-Y reconstruction compared with Billroth II partial gastrectomy. *Am J Surg.* 1988;156(1):21-25.

5. Suzuki H, Iijima K, Moriya A, et al. Conditions for acid catalysed luminal nitrosation are maximal at the gastric cardia. *Gut.* 2003;52(8):1095-1101.

6. Verdu E, Viani F, Armstrong D, et al. Effect of omeprazole on intragastric bacterial counts, nitrates, nitrites, and N-nitroso compounds. *Gut.* 1994;35(4):455-460.

7. Vermeer IT, van Maanen JM. Nitrate exposure and the endogenous formation of carcinogenic nitrosamines in humans. *Rev Environ Health.* 2001;16(2):105-116.

8. Walters CL, Carr FP, Dyke CS, Saxby MJ, Smith PL, Walker R. Nitrite sources and nitrosamine formation in vitro and in vivo. *Food Cosmet Toxicol.* 1979;17(5):473-479.

9. Watt PC, Sloan JM, Donaldson J, Campbell G, Kennedy TL. Relation between gastric histology and gastric juice pH and nitrite and N-nitroso compound concentrations in the stomach after surgery for duodenal ulcer. *J Clin Pathol.* 1984;37(5):511-515.

10. De Bernardinis G, Guadagni S, Pistoia MA, et al. Gastric juice nitrite and bacteria in gastroduodenal disease and resected stomach. *Tumori.* 1983;69(3):231-237.

11. Guadagni S, Walters CL, Smith PL, Verzaro R, Valenti M, Reed PI. N-nitroso compounds in the gastric juice of normal controls, patients with partial gastrectomies, and gastric cancer patients. *J Surg Oncol.* 1996;63(4):226-233.

12. Ruddell WS, Bone ES, Hill MJ, Blendis LM, Walters CL. Gastric-juice nitrite. A risk factor for cancer in the hypochlorhydric stomach? *Lancet.* 1976;2(7994):1037-1039.

13. Ruddell WS, Bone ES, Hill MJ, Walters CL. Pathogenesis of gastric cancer in pernicious anaemia. *Lancet.* 1978;1(8063):521-523.

14. Sobala GM, Pignatelli B, Schorah CJ, et al. Levels of nitrite, nitrate, N-nitroso compounds, ascorbic acid and total bile acids in gastric juice of patients with and without precancerous conditions of the stomach. *Carcinogenesis.* 1991;12(2):193-198.

15. Schlag P, Bockler R, Ulrich H, Peter M, Merkle P, Herfarth C. Are nitrite and N-nitroso compounds in gastric juice risk factors for carcinoma in the operated stomach? *Lancet.* 1980;1(8171):727-729.

16. Schlag P, Bockler R, Peter M. Nitrite and nitrosamines in gastric juice: risk factors for gastric cancer? *Scand J Gastroenterol.* 1982;17(1):145-150.

17. Stockbrugger RW, Cotton PB, Eugenides N, Bartholomew BA, Hill MJ, Walters CL. Intragastric nitrites, nitrosamines, and bacterial overgrowth during cimetidine treatment. *Gut.* 1982;23(12):1048-1054.

© 2021 Gao Z et al. *JAMA Network Open.*

18. Tari A, Kodama K, Sumii M, Tani H, Sumii K, Chayama K. Does intragastric nitrite concentration reflect gastric carcinogenesis in Japanese Helicobacter pylori-infected patients? *Dig Dis Sci.* 2003;48(9):1730-1736.

19. Kyrtopoulos SA, Daskalakis G, Legakis NI, et al. Studies in gastric carcinogenesis. II. Absence of elevated concentrations of N-nitroso compounds in the gastric juice of Greek hypochlorhydric individuals. *Carcinogenesis.* 1985;6(8):1135-1140.

20. Watt PC, Sloan JM, Donaldson JD, Patterson CC, Kennedy TL. Relationship between histology and gastric juice pH and nitrite in the stomach after operation for duodenal ulcer. *Gut.* 1984;25(3):246-252.

21. Vu BD, Paul JL, Gaudric M, Guerre J, Yonger J, Ekindjian OG. N-nitroso compounds, nitrite and pH in human fasting gastric juice. *Carcinogenesis.* 1994;15(11):2657-2659.

22. Thorens J, Froehlich F, Schwizer W, et al. Bacterial overgrowth during treatment with omeprazole compared with cimetidine: a prospective randomised double blind study. *Gut.* 1996;39(1):54-59.

23. Pignatelli B, Malaveille C, Rogatko A, et al. Mutagens, N-nitroso compounds and their precursors in gastric juice from patients with and without precancerous lesions of the stomach. *Eur J Cancer.* 1993;29A(14):2031-2039.

24. Xu GP, Reed PI. N-nitroso compounds in fresh gastric juice and their relation to intragastric pH and nitrite employing an improved analytical method. *Carcinogenesis.* 1993;14(12):2547-2551.

25. Walters CL, Dyke CS, Saxby MJ. Nitrosation of food amines under stomach conditions. *IARC Sci Publ.* 1976(14):181-193.

26. Hall CN, Darkin D, Brimblecombe R, Cook AJ, Kirkham JS, Northfield TC. Evaluation of the nitrosamine hypothesis of gastric carcinogenesis in precancerous conditions. *Gut.* 1986;27(5):491-498.

27. Keighley MR, Youngs D, Poxon V, et al. Intragastric N-nitrosation is unlikely to be responsible for gastric carcinoma developing after operations for duodenal ulcer. *Gut.* 1984;25(3):238-245.

28. Thomas JM, Misiewicz JJ, Cook AR, et al. Effects of one year's treatment with ranitidine and of truncal vagotomy on gastric contents. *Gut.* 1987;28(6):726-738.

© 2021 Gao Z et al. *JAMA Network Open.*
